# Oral corticosteroid overexposure: characterizing oral corticosteroid use in patients with chronic rhinosinusitis with nasal polypS in Canada (ACTIONS) results

**DOI:** 10.1186/s13223-025-00989-w

**Published:** 2025-12-04

**Authors:** Yvonne Chan, Marie-Noëlle Corriveau, Jeffrey Beach, Jenna Reynolds, Koyo Usuba, Danya Thayaparan, Ali Tehrani, Juejing Ling, Huijuan Yang, Andrew Thamboo

**Affiliations:** 1 Department of Otolaryngology - Head & Neck Surgery, St. Michael’s Hospital, University of Toronto, Toronto, ON Canada; 2 CHU de Québec, Université Laval, Quebec City, QC Canada; 3 Asthma Canada, Toronto, ON Canada; 4 Health Economics and Outcomes Research, GSK, Mississauga, ON Canada; 5 Medical Affairs, GSK, Mississauga, ON Canada; 6 IQVIA Solutions Canada, Inc., Kirkland, QC Canada; 7 St. Paul’s Hospital, 1081 Burrard St, Vancouver, BC V6Z 1YT Canada

**Keywords:** Asthma, Chronic disease, Chronic rhinosinusitis, Corticosteroid use, Medical therapy of chronic rhinosinusitis

## Abstract

**Background:**

Chronic rhinosinusitis with nasal polyps (CRSwNP) is a type 2 inflammatory condition, often treated with oral corticosteroids (OCS). OCS overexposure can increase the risk of adverse effects, highlighting the need for better treatment strategies including timely biologic therapy use. This study explored OCS use patterns in patients with severe CRSwNP in Canada pre-biologic initiation.

**Methods:**

This retrospective, real-world study used IQVIA’s Private Drug Plan (PDP) claims database to examine OCS use patterns 0–24 months pre-initiation of biologic therapy in patients with inferred CRSwNP in Canada (*n* = 747). Outcomes included OCS use patterns (proportions, claims per patient and by prescribing physician specialty, overexposure [≥ 1000 mg annually, prednisone-equivalent]), demographics, and stratified by comorbid asthma.

**Results:**

Of 747 patients indexed from the PDP, 81.0% had ≥ 1 OCS claim(s), with a median (interquartile range [IQR]) of 3.0 (4.0) claims per patient. Median (IQR) total OCS dose over 24 months was 1020 (1420) mg; 41.5% of patients experienced OCS overexposure. OCS was primarily prescribed by general practitioners (35.9%) and otolaryngologists (32.8%). Patients had a median (IQR) age of 51 (18) years at index, 52.1% were male, and most were from Ontario (48.7%). OCS use was highest in patients with comorbid severe asthma.

**Conclusions:**

This study highlights OCS overexposure in patients with CRSwNP pre-biologic initiation. Enhancing physician and patient awareness of appropriate OCS use and timely biologic initiation could reduce the risk of OCS-related adverse effects and improve CRSwNP management, benefiting long-term patient outcomes through shared decision-making.

*Trial registration* GSK study number 221872.

**Supplementary Information:**

The online version contains supplementary material available at 10.1186/s13223-025-00989-w.

## Introduction

Chronic rhinosinusitis with nasal polyps (CRSwNP) is a type 2 inflammatory subtype of chronic rhinosinusitis affecting the nasal cavities and paranasal sinuses, characterized by benign inflammatory masses that develop bilaterally in the sinonasal cavity [1, 2]. CRSwNP symptoms include nasal blockage and discharge, reduced or loss of smell, and/or pressure or pain in the face [1, 2], which can cause depression and sleep problems [3], altogether conferring a negative impact on quality of life. Typical comorbidities include asthma (prevalence ranging from 5–56%), allergies (12–77%), and allergic rhinitis (17–76%) [4]. In Canada, chronic rhinosinusitis affects 3.4–5.7% of individuals [5], although data are lacking on the prevalence of CRSwNP specifically [4]. Approximately 1.2% of the United States (US) population have CRSwNP [6].

CRSwNP is typically managed using saline irrigation and intranasal corticosteroids (INCS) [2]. Severe CRSwNP is characterized by bilateral obstructive polyps, extensive involvement of the sinuses, and persistent symptoms despite long-term INCS treatment [7]. Short-term use of systemic or oral corticosteroids (OCS), which may be referred to as an “OCS burst” [8], can be added to first-line therapy to improve symptoms and nasal polyp size [2]; however, longer-term or frequent use is not recommended due to the increased risk of adverse effects [9, 10]. In patients who have recalcitrant CRSwNP and are unresponsive to medical intervention, endoscopic sinus surgery is considered, but many require revision surgery due to disease recurrence [11, 12]. It is therefore essential to consider alternative management options, including biologic therapy, for patients whose CRSwNP remains uncontrolled despite medical and surgical intervention.

OCS use, and in particular cumulative use over a prolonged period of time, is associated with an increased risk of adverse effects; hence, it should be used sparingly and is recommended as a last resort after combinations of other medications prove ineffective [13, 14]. Cumulative or lifetime OCS exposures of 1000–2500 mg (and for some outcomes from 500–1000 mg) have been associated with risk of adverse effects such as osteoporosis, pneumonia, cardiovascular or cerebrovascular diseases, type 2 diabetes mellitus, cataracts, sleep apnea, renal impairment, depression or anxiety, and weight gain [10]. Therefore, receipt of a cumulative or lifetime dose of > 1000 mg OCS is considered overexposure [10]. Furthermore, adverse effects of OCS in adults such as osteoporosis, hyperglycemia, and muscle weakness have been associated with short-term use of < 7 days and cumulative exposures as low as 500– < 1000 mg [15]. Clinicians surveyed on the use of OCS for CRSwNP typically considered an annual OCS dose of 800–2000 mg, with a treatment duration ranging from 4 to 8 weeks, increased the risk of adverse effects, indicating a potential knowledge gap to be bridged between research and clinical practice in terms of what is considered “too much” OCS [13]. In addition, in a retrospective US study including > 20,000 patients with CRSwNP, 64.7% of patients received systemic corticosteroids within 12 months, with 23.5% receiving > 400 mg of the OCS prednisone without consideration of the severity of the disease [8]. Inappropriate use of OCS appears to be widespread yet [8, 16], to our knowledge, there are no data to quantify the utilization of OCS for patients with CRSwNP in Canada.

To date, three biologic therapies have been approved in Canada for the treatment of severe CRSwNP [17]: dupilumab (2020) [18], omalizumab (2021) [19], and mepolizumab (2021) [20], all of which have been associated with improved outcomes, such as decreased Sinonasal Outcome Test-22 scores, in clinical trials and real-world studies for CRSwNP [21, 22]. Furthermore, all are approved for the treatment of severe asthma, a common comorbidity of CRSwNP, and have been shown to improve outcomes in patients with CRSwNP and comorbid severe asthma [23–25].

The retrospective ChAracterizing uncontroLled sevERe asThma in Canada (ALERT) study, using longitudinal claims data from IQVIA’s Private Drug Plan (PDP) database (2020–2023), found that the majority of patients with uncontrolled severe asthma had no biologic therapy claims but had substantial OCS use [26]. This highlights the overexposure to OCS in patients with asthma and raises the question of whether similar patterns are prevalent in patients with CRSwNP – an important consideration due to the risks associated with OCS overexposure [13, 14].

The aim of this study was to describe patterns of OCS use among patients with severe CRSwNP in Canada, pre-initiation of biologic therapy.

## Methods

### Study design

The chAracterizing oral Corticosteroid use in patients wiTh chronic rhInOsinusitis with Nasal polypS in Canada (ACTIONS) study was a retrospective, real-world study aiming to describe the pattern of OCS use pre-initiation of biologic therapy for CRSwNP treatment, utilizing IQVIA’s PDP claims database, the largest national private claims database in Canada. The included suppliers represent approximately 80% of all pay-direct private plan claims and > 12 million active claimants with > 129 million drug claims in Canada [27]. Patients were identified in the database between August 12, 2020–November 30, 2023 (Supplementary Fig. 1). The index date was defined as the date of the first claim of a biologic therapy of interest (dupilumab, mepolizumab, or omalizumab) initiated for CRSwNP. The 0–24 months pre-index, designated as the “analysis period”, was used to describe OCS use pre-initiation of biologic therapy for CRSwNP. The 3 months prior to the analysis period was used to ensure patients were active in the database i.e., that they had ≥ 1 claim(s) in any market.

### Study population

Eligible patients had ≥ 1 claim(s) for initiation of a biologic therapy of interest (dupilumab, mepolizumab, or omalizumab) in the selection period, were aged ≥ 18 years at index, and had inferred CRSwNP, identified as follows. Rule-based inference algorithms, which were validated by experts (Y.C. and A.T.) during protocol development, were used to infer patients with a diagnosis of CRSwNP, as depicted in Supplementary Fig. 2. This process was based on patient drug claims history and physician specialty. For patients who did not meet the initial CRSwNP diagnosis criteria, the physician specialty for the index biologic therapy claim was assessed, and patients were inferred as having CRSwNP if the index biologic therapy was prescribed by an otolaryngologist. Patients who were inferred as having CRSwNP or CRSwNP with comorbid severe asthma (≥ 1 other biologic prescription in the 2 years prior; or ≥ 1 inhaled corticosteroids/long-acting β2-agonist and ≥ 1 leukotriene receptor antagonist and ≥ 8 total prescriptions of any respiratory products and have used ≥ 4 different products 12 months before the last respiratory or biologic prescription, and had a history of asthma-only biologics) and/or atopic dermatitis (patients with ≥ 1 marker of atopic dermatitis [topical corticosteroids, crisaborole or topical calcineurin inhibitors] in the 2 years prior to index) were included in this study. Patients were excluded if they had any claim for a biologic therapy (including those of interest) pre-index or were not active in the database i.e., with no claims in the 3 months prior to the analysis period, to ensure continuous activity throughout the analysis period. As noted, biologic therapies of interest included those approved for use in severe CRSwNP (dupilumab, mepolizumab, and omalizumab) in addition to those approved for use in severe asthma, which were additionally selected to screen for biologic-naïvety (benralizumab, reslizumab, and tezepelumab); although these biologics are not yet approved for CRSwNP, they were excluded to eliminate any potential confounding effects.

### Study objectives

The primary objective was to describe the pattern of OCS use pre-initiation of biologic therapy for treatment of CRSwNP in patients with inferred CRSwNP in Canada. This included: the proportions of patients with OCS use; OCS claims per patient; OCS claims by prescribing physician specialties; dose, duration, and frequency of OCS use; and the proportion of patients with OCS overexposure (defined as a total annual dose ≥ 1000 mg, prednisone/equivalent). The secondary objective was to describe the demographics among patients with inferred CRSwNP in Canada.

Exploratory objectives included describing, where feasible, the demographics and pattern of OCS use for asthma sub-cohorts (mild/moderate or severe asthma based on prescriptions for inhaler therapy based on the ALERT study) [26]. Also assessed as exploratory objectives were the demographics and patterns of OCS use in different OCS sub-cohorts (according to short-term “OCS burst” use [3–28 days supplied of ≥ 20 mg OCS prednisone/equivalent] or longer-term “OCS maintenance” use [> 28 days supplied]), and in the total study cohort at an appropriate sub-provincial level (described in the Supplementary Material).

### Study analyses

Outcomes for patients with inferred CRSwNP were derived based on the drug claims in patient claims history in the databases retrospectively within the 24-month analysis period pre-index initiation of biologic therapy, and results are presented according to the order of study objectives. Demographic variables were reported based on the index date and within the analysis period, and included: biological sex; age; Canadian provincial geography; presence of comorbid inferred atopic dermatitis; polypharmacy (the number of concomitant medication classes taken); antibiotic use; inhaler use for asthma; and INCS use.

Outcomes associated with OCS use were reported over the 24-month analysis period, in the 0–12 months and the 13–24 months pre-index, derived from drug claims available in the database. These outcomes included the number and proportion of patients with OCS use and the total number of OCS claims per patient. Among patients with ≥ 1 claim(s) for OCS, the following data were evaluated: the number of claims (including by prescribing physician specialties for OCS per patient); the duration and frequency (total number of days supplied for OCS per patient and average number of days between claims); and dose (total dose and total daily dose of OCS per patient, number, and proportion of patients by OCS dose and with OCS overexposure). Daily dose was defined as total milligrams of OCS per day, calculated based on the total OCS milligrams (prednisone/equivalent) divided by the total number of days supplied prescribed within the analysis period. OCS overexposure was defined as a total annual OCS dose ≥ 1000 mg (prednisone/equivalent), to account for the fixed study period of 2 years. All values with < 6 patients or claims were masked by attributing a value of 3* to ensure anonymity.

All data analyses were conducted in accordance with IQVIA standard operating procedures. Descriptive statistics were reported as median (interquartile range) for continuous variables, and number and proportion for categoric variables. Missing data were reported as a separate category and no imputation was performed. All analyses were conducted using SAS Enterprise Guide (SAS Institute, Cary, NC, USA) version 6.1 or higher and Microsoft Excel.

### Ethical approval

This study is based on the analysis of de-identified patients in the IQVIA claims databases and confidentiality of patient records was maintained at all times. Study results were presented in aggregated tabular form that omits patient identification. A waiver of informed consent was not required for this study, nor was institutional review board or ethical approval as the source data were anonymized.

## Results

### OCS use pre-initiation of biologic therapy for treatment of CRSwNP

**Fig. 1:**
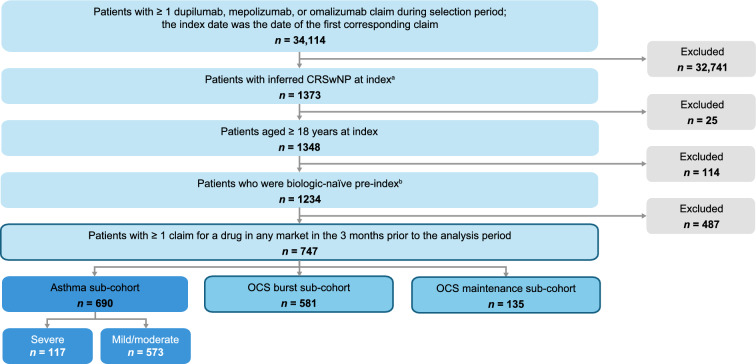
Patient selection from PDP database. ^a^Indication of CRSwNP was inferred based on three rule-based algorithms with claims for dupilumab, mepolizumab, and omalizumab recorded in the claims databases. These algorithms used patient claims history and prescribing physician specialty, where available and were developed by GSK and IQVIA. ^b^Patients who had no claims for a biologic therapy of interest pre-index; the biologic therapies of interest included dupilumab, mepolizumab, omalizumab, benralizumab, reslizumab, and tezepelumab. CRSwNP, chronic rhinosinusitis with nasal polyps; OCS, oral corticosteroids; PDP, Private Drug Plan

**Fig. 2:**
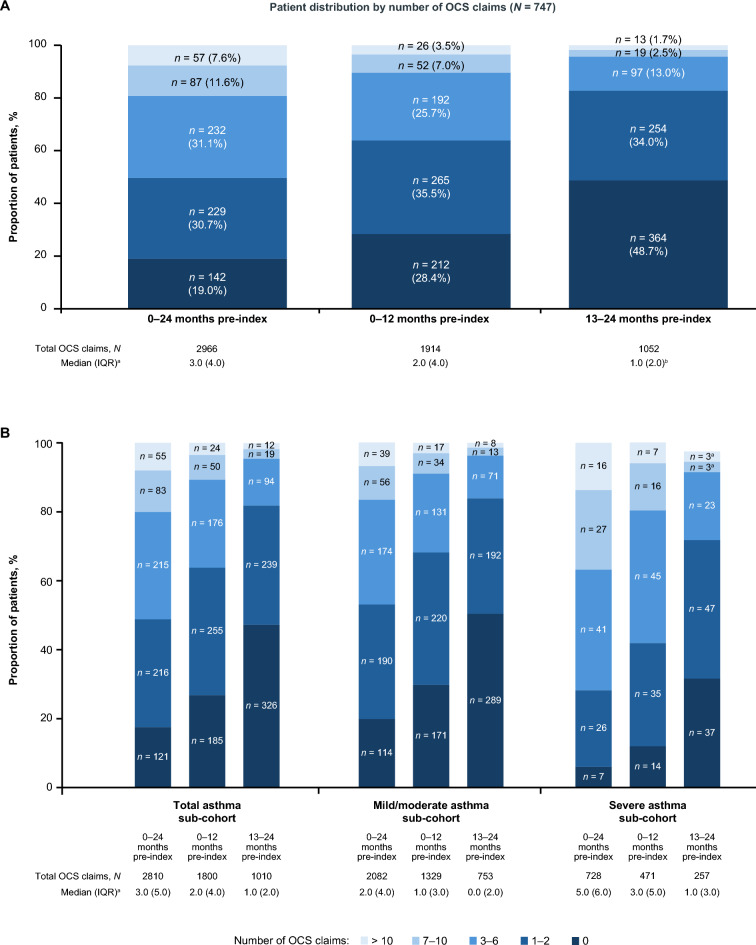
Proportion of patients with OCS claims and median (IQR) OCS dose of all patients in the total population (**A**) and asthma sub-cohorts (**B**). ^a^All values with a count of less than six patients or claims were masked as three according to privacy rules including secondary values. IQR, interquartile range; OCS, oral corticosteroids

In total, 747 patients were eligible for inclusion in the study (Fig. 1). Over the 24-month analysis period, 605 (81.0%) patients had ≥ 1 claim(s) for OCS and 376 (50.3%) patients had > 2 OCS claims, with a median (IQR) of 3.0 (4.0) OCS claims per patient amongst all patients (Fig. 2A). Most (96.4%) OCS claims were for prednisone.

**Fig. 3:**
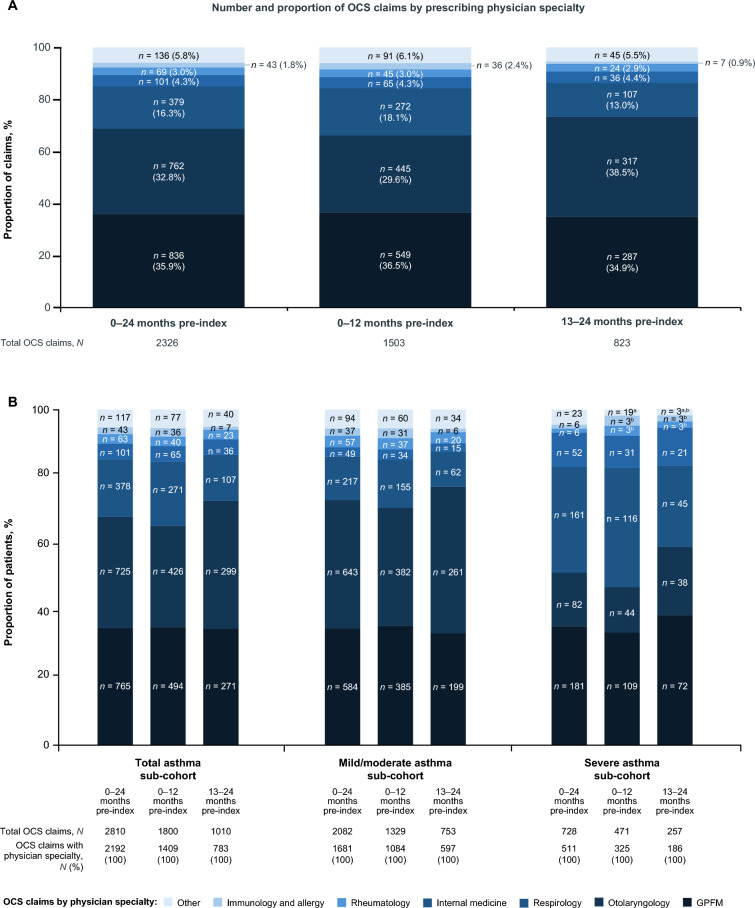
Proportion of OCS claims by physician specialty in the total population (**A**) and asthma sub-cohorts (**B**). ^a^Calculated with secondary masking to prevent back calculation. ^b^All values with a count of less than six patients or claims were masked as three according to privacy rules including secondary values. GPFM, general practice and family medicine; OCS, oral corticosteroids

In the total population, across the analysis period, most OCS claims were prescribed by general practice and family medicine (GPFM) practitioners (35.9%), otolaryngologists (32.8%), and respirologists (16.3%). The median (IQR) daily dose of OCS per claim among patients with ≥ 1 claim(s) over the full 2 years prior to index (0–24 months) was highest among GPFM practitioners at 37.5 (30.0) mg, followed by respirologists at 31.0 (20.0) mg, and otolaryngologists at 30.0 (19.3) mg. Claim distributions in the 0–12 and 13–24 months pre-index were comparable to that of the full analysis period (Fig. 3A). For 45.1% of patients, their OCS claims were prescribed by one physician specialty only, while 27.3% of patients had OCS claims from > 1 physician specialty (median [IQR] number of physician specialties for OCS claims per patient, 1.0 [1.0]).

**Fig. 4:**
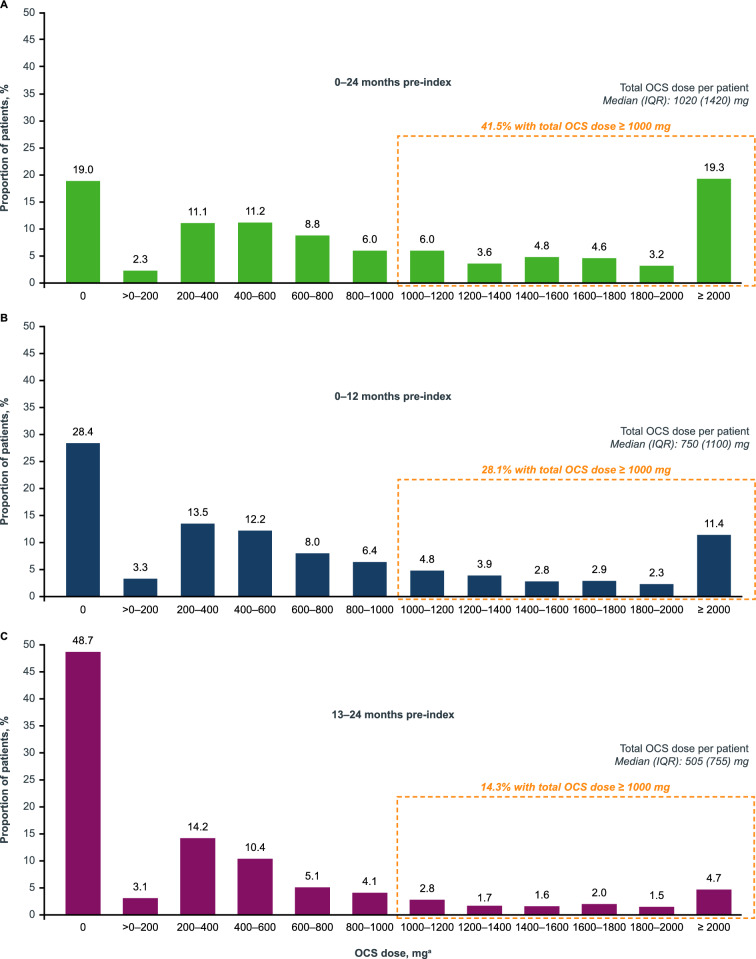
OCS doses received by patients in the total population in the full (**A**) 24-month analysis period, (**B**) 0–12 months pre-biologic therapy initiation, and (**C**) 13–24 months pre-biologic therapy initiation. ^a^Dose range includes the lower bound in the interval, but not the upper bound. IQR; interquartile range; OCS, oral corticosteroids.

Over the 2-year analysis period, patients received a total median (IQR) dose of 1020 (1420) mg OCS. Total median (IQR) dose was 750 (1100) mg and 505 (755) mg for 0–12 months and 13–24 months pre-biologic therapy initiation, respectively (Fig. 4A–C). Overall, 41.5% of patients received ≥ 1000 mg in the 2-year analysis period (including patients who were overexposed within either 1 year or 2 years pre-biologic therapy) compared with 28.1% and 14.3% for 0–12 months and 13–24 months pre-biologic therapy initiation, respectively. On average, patients in the overall cohort had approximately 2.5 months’ supply of OCS over the analysis period, with 32 mg per day, and only 18.1% had a daily dose of OCS of ≤ 20 mg.

### Demographics in the overall population

Patient demographics and clinical characteristics are presented in Table 1. Patients had a median (IQR) age of 52 (18) years at index, 52.1% were male, and most were from Ontario (48.7%) or Quebec (28.5%). Overall, 86.9% of patients had ≥ 5 concomitant therapeutic classes prescribed during the study period. The most commonly claimed concomitant therapeutic classes were anti-asthma and chronic obstructive pulmonary disease (COPD) therapies (92.9%), systemic corticosteroids (82.2%), systemic antibacterials (79.4%), and nasal preparations (73.9%).

**Table 1:** Demographic characteristics of total study cohort and asthma sub-cohorts

	Total study cohort (*N* = 747)	Asthma sub-cohorts		
		Total asthma (*n* = 690)	Severe (*n* = 117)	Mild/moderate (*n* = 573)
Median age, years (IQR)	52 (18)	52 (17)	56 (15)	52 (18)
Biological sex, n (%)^a^				
Male	389 (52.1)	357 (51.7)	65 (55.6)	292 (51.0)
Female	355 (47.5)	330 (47.8)	50 (42.7)	280 (48.9)
Provinces in PDP database, n (%)				
Alberta	66 (8.8)	59 (8.6)	8 (6.8)	51 (8.9)
British Columbia	26 (3.5)	23 (3.3)	3^b^	22 (3.8)
Manitoba	25 (3.3)	23 (3.3)	3^b^	19 (3.3)
New Brunswick	20 (2.7)	19 (2.8)	9 (7.7)	10 (1.7)
Newfoundland & Labrador	3^b^	3^b^	0 (0.0)	3^b^
Nova Scotia	21 (2.8)	18 (2.6)	3^b^	15 (2.6)
Ontario	364 (48.7)	336 (48.7)	49 (41.9)	287 (50.1)
Quebec	213 (28.5)	200 (29.0)	43 (36.8)	157 (27.4)
Saskatchewan	3^b^	3^b^	0 (0.0)	3^b^
Presence of comorbid AD, n (%)				
Yes	88 (11.8)	82 (11.9)	3^b^	77 (13.4)
No	659 (88.2)	608 (88.1)	112 (95.7)	496 (86.6)
Medication use, n (%)				
Antibiotic use	594 (79.5)	566 (82.0)	102 (87.2)	464 (81.0)
Inhaler use	680 (91.0)	678 (98.3)	117 (100)	561 (97.9)
INCS use	506 (67.7)	474 (68.7)	94 (80.3)	380 (66.3)
Polypharmacy category, n (%)^c^				
1–4	91 (12.2)	78 (11.3)	3^b^	70 (12.2)
5–7	204 (27.3)	192 (27.8)	22 (18.8)	170 (29.7)
8–10	185 (24.8)	172 (24.9)	28 (23.9)	144 (25.1)
11–13	143 (19.1)	138 (20.0)	31 (26.5)	107 (18.7)
14–16	65 (8.7)	61 (8.8)	12 (10.3)	49 (8.6)
17–19	30 (4.0)	29 (4.2)	11 (9.4)	18 (3.1)
20 +	22 (2.9)	20 (2.9)	3^b^	15 (2.6)
Unknown	7 (0.9)	0 (0)	0 (0)	0 (0)

AD, atopic dermatitis; (Eph)ATC, (European Pharmaceutical) Anatomical Therapeutic Chemical classification; INCS, intranasal corticosteroids; IQR, interquartile range; PDP, Private Drug Plan

^a^Sum of values may not equal 100% as some data were unknown

^b^All values with a count of less than six patients or claims were masked as three according to privacy rules including secondary values

^c^Polypharmacy was defined as the number of EphATC (level 2) classes in any market in the 24-month analysis year; patients with unknown polypharmacy had the ATC information unavailable in the 24-month analysis period

### Demographics and patterns of OCS use in different asthma sub-cohorts

Overall, 690 patients were included in the total asthma sub-cohort. Among them, 573 patients (83.0%) were in the mild/moderate asthma sub-cohort and 117 (17.0%) were in the severe asthma sub-cohort. The characteristics of the total asthma sub-cohort were comparable to the overall study population, except for a numerically slightly higher use of antibiotics, inhalers, and INCS that were observed in 82.0%, 98.3%, and 68.7% of patients, respectively (Table 1).

In the total asthma sub-cohort: 569 (82.5%) of patients had ≥ 1 OCS claim(s), with a median (IQR) of 3.0 (4.0) OCS claims per patient with ≥ 1 claim(s) (Fig. 2B). The severe asthma sub-cohort had greater use of OCS compared with the mild/moderate asthma sub-cohort, with a median (IQR) of 5.0 (5.0) versus 3.0 (4.0) OCS claims per patient with ≥ 1 claim(s), respectively.

Similar trends in prescribing physician specialties were seen in the total asthma sub-cohort compared with the overall study population (Fig. 3B). In the mild/moderate asthma sub-cohort, most OCS claims were prescribed by otolaryngologists (38.3%), followed by GPFM (34.7%) and respirologists (12.9%), whereas in the severe asthma sub-cohort most claims came from GPFM (35.4%), respirologists (31.5%), and otolaryngologists (16.0%). The median (IQR) number of physician specialties for OCS claims per patient was 1.0 (1.0) in the mild/moderate asthma sub-cohort and 2.0 (1.0) in the severe asthma sub-cohort.

**Fig. 5:**
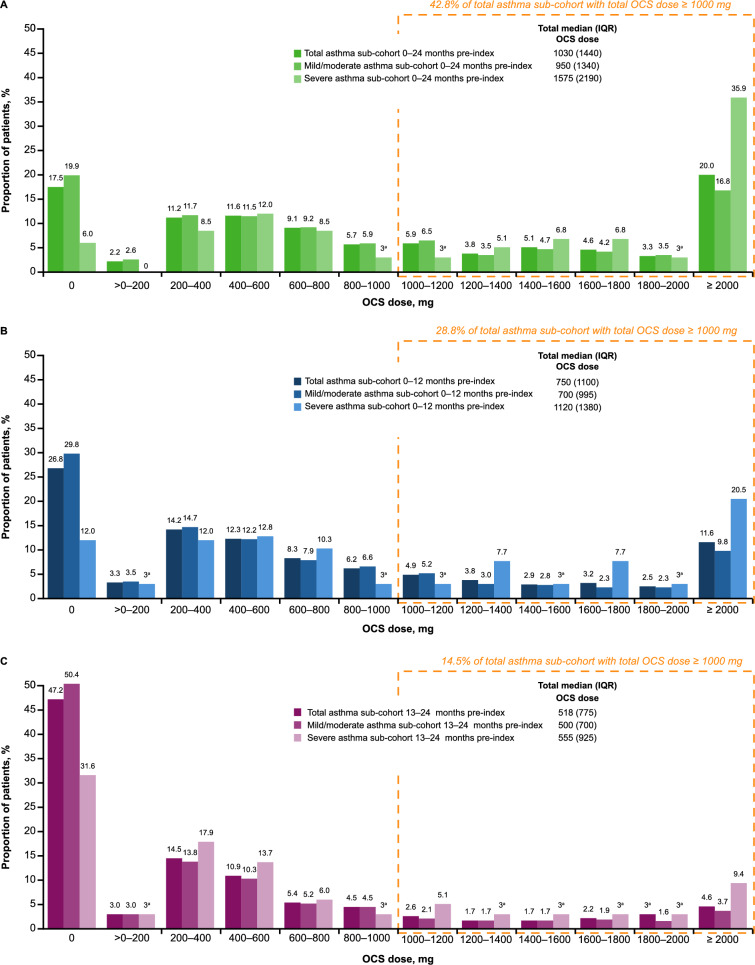
OCS doses received by patients in the asthma sub-cohort from (**A**) 0–24 months, (**B**) 0–12 months, and (**C**) 13–24 months pre-index. ^a^All values with a count of less than six patients or claims were masked as three according to privacy rules including secondary values. ^†^Dose range includes the lower bound in the interval, but not the upper bound. IQR, interquartile range; OCS, oral corticosteroids

A similar pattern in OCS use was seen with the total asthma sub-cohort compared with the overall study population. Over the analysis period, a higher total OCS dose was observed in the severe asthma sub-cohort compared with the mild/moderate asthma sub-cohort, with a median (IQR) total OCS dose of 1575 (2190) mg versus 950 (1340) mg, respectively (Fig. 5A). OCS overexposure in the 0–12 months, 13–24 months, or full analysis period pre-index was observed in 56.4% of patients in the severe asthma sub-cohort versus 38.1% of patients in the mild/moderate asthma sub-cohort.

### Demographics and patterns of OCS use in “OCS burst” and “OCS maintenance” sub-cohorts and at sub-provincial level

When categorized according to duration of OCS use, 581 patients and 135 patients were included in the OCS burst and OCS maintenance sub-cohorts, respectively (Fig. 1); their demographic characteristics were generally comparable between the two sub-cohorts (Supplementary Table 1). The OCS maintenance sub-cohort had more OCS claims and higher doses than the OCS burst sub-cohort. Further details of OCS dose, duration, and overexposure are presented in the supplement (Supplementary Fig. 3–5). Patients in the OCS burst sub-cohort had similar patterns of OCS claims by physician specialty as the total study population, while patients in the OCS maintenance sub-cohort had a lower proportion of OCS claims prescribed from otolaryngologists and a higher proportion from respirologists (Supplementary Fig. 6).

Data regarding the OCS use pattern at a sub-provincial level are provided in the Supplementary Results (Supplementary Table 2).

## Discussion

This study addresses a current gap in the literature by investigating OCS use pre-biologic therapy initiation in patients with severe CRSwNP in Canada. It is important to note that the patients included in this study ultimately required biologic therapy, indicating that their symptoms were not adequately controlled by medical or surgical intervention alone, leading to high CRSwNP disease severity and burden. This is evidenced by the high OCS use observed in this study. Over the 24-month analysis period, 81.0% of all patients had ≥ 1 OCS claim(s) and 50.3% had > 2 OCS claims, with a median of 3.0 OCS claims per patient, equating to a median total OCS dose (prednisone/equivalent) of 1020 mg.

Specifically, patients tended to have a higher total OCS dose in the 0–12 months pre-index than in the 13–24 months pre-index. This implies that the overall prevalence of OCS use before initiating biologic therapy is high and it is likely that patients require higher-dose OCS to manage symptoms due to disease progression until they initiate biologic therapy. Such findings are supported by a US-based retrospective real-world study which found that prior OCS treatment was a predictive factor for biologic therapy use in patients with CRSwNP [28]. As biologic therapies reduce the use of OCS in patients with CRSwNP [29], it is essential to realize the extent of the OCS claims in this population to prevent important adverse effects by initiating biologic therapies earlier in the treatment course. The ACTIONS study clearly demonstrates high OCS use among patients with CRSwNP and comorbid asthma, reinforcing the need to initiate biologic therapy sooner. However, access to biologics in Canada remains challenging, and surgery continues to be a more cost-effective and accessible treatment option for most patients with CRSwNP [11]. Eligibility of biologics is restricted to patients with severe symptoms, and typically only after failure of conventional therapies such as INCS and surgery [11]. In Canada’s single-payer healthcare system, biologics are often funded through private pharmaceutical insurance and their high cost, ranging from CAD$600 to CAD$4000 per vial or syringe (2017–2020), limits widespread use [11, 30, 31]. Consequently, biologics are generally reserved for select patients where other options have been exhausted [11]. These barriers to access may contribute to continued reliance on OCS, despite the known risks of overexposure.

Among patients with OCS claims, 41.5% were overexposed to OCS within either 0–12 or 13–24 months pre-index (39.3% and 27.9%, respectively). We expect that the proportion of patients exceeding the recommended lifetime dose of 1000 mg (prednisone/equivalent) may be even higher than this as many patients may continue to experience cumulative OCS exposure beyond the 2-year window examined in our study. OCS overexposure in the 0–12 months, 13–24 months, or both pre-index was also observed in more patients with severe asthma (56.4%) versus mild/moderate asthma (38.1%). The prevalence of comorbid asthma in this study is comparable to other real-world studies of biologic therapies in severe CRSwNP [21, 29]. Patients in the severe asthma sub-cohort reported higher OCS use than the mild/moderate asthma sub-cohort, with 5.0 versus 3.0 OCS claims per patient among those with ≥ 1 claim(s), respectively. These results are reflective of the wider literature that reveal higher OCS use among patients with severe asthma than those with mild/moderate asthma [32, 33]. Furthermore, this is similar to the retrospective ALERT study which found that the majority (72%) of patients with uncontrolled severe asthma (*n* = 11,208) had no biologic therapy claims but had substantial OCS use (mean 4.4 OCS claims per patient; 8% of patients had ≥ 10 OCS claims per year) [26]. Together with the results from previous studies, which reported that the risk of OCS-related adverse effects is associated with higher cumulative or lifetime OCS exposure [10, 34], this study highlights the prevalence of OCS overexposure and thus the importance of improving awareness of the potential risk of OCS overexposure among patients.

When examining the prescribing physicians for OCS claims, 35.9% of OCS claims in this study were prescribed by GPFM, followed by otolaryngologists (32.8%). This is consistent with a previous study which reported that family practices were the top physician specialty most frequently (31.6%) prescribing OCS among the general population in the US [35]. However, it is noteworthy that the US study investigated OCS patterns in a general population; 16.5% of the short-course OCS prescriptions were indicated for COPD, asthma, and other respiratory conditions, compared with our study which investigated patients with CRSwNP. In a recent survey of clinicians in Italy, 20.3% of surveyed otorhinolaryngologists (*n* = 437) responded that they would prescribe OCS for CRSwNP regardless of the number of cycles per year [13], suggesting that some guidance may be better targeted at specialties who treat patients with CRSwNP most frequently. In addition, 27.3% of all patients with ≥ 1 claim(s) obtained OCS from ≥ 2 physician specialties, and the percentage was higher in the OCS maintenance sub-cohort (39.3%). This suggests better patient–physician or physician–physician communication may help to avoid OCS overexposure.

A strength of this study is the large size of the dataset, with 747 eligible patients included, from 34,114 patients having ≥ 1 claim(s) for dupilumab, mepolizumab, or omalizumab therapy. A limitation is that the diagnosis was inferred by the rule-based algorithm using patient claims history and prescribing physician specialty. The claims database did not contain CRSwNP diagnosis or severity information. Furthermore, this methodology may have introduced a misclassification bias as some of the medications used in the algorithm may have had indications in addition to CRSwNP and therefore patients with other severe diseases may have been included. These two limitations may have therefore led to over- or under-estimation of the number of patients with a CRSwNP diagnosis; however, the results from ACTIONS are similar to a prior estimate of OCS use in patients with CRSwNP. ACTIONS reports that 71.6% of patients with severe CRSwNP received OCS in the 12 months pre-index period; a retrospective US study of > 20,000 patients reported that 64.7% of patients with CRSwNP across severities received systemic corticosteroids within 12 months pre-diagnosis. Another limitation is that OCS use in our study could not be limited to CRSwNP-specific claims, and overall OCS use may be underreported considering this study examined PDPs and did not include OCS use in patients covered by public plans. Additionally, since this study focused on the analysis of OCS claims from PDPs, information regarding adverse effects from OCS use and the number of patients who had undergone surgery was not available. Furthermore, given the high prevalence of comorbid asthma [4], OCS overexposure may occur as a result of treating both CRSwNP and asthma [26]. This overlap presents a challenge in attributing OCS use solely to CRSwNP, particularly as more frequent or higher-dose OCS use may be needed in severe cases or for comorbid asthma management. In our cohort, 92% of patients had comorbid asthma and 16% had severe asthma, which may introduce bias in interpreting OCS burden and treatment escalation needs for CRSwNP. Nevertheless, patients with uncontrolled, severe CRSwNP rarely present without accompanying comorbidities; it has been shown that severe, uncontrolled CRSwNP is frequently associated (up to 67%) with comorbid asthma, contributing to greater disease severity and an increased requirement for biologic therapy than CRSwNP without comorbid asthma, which aligns with the population observed in this study [36]. Improved physician–physician communication and patient education could optimize patient care, particularly for those with comorbidities, and reduce OCS use. Finally, as the study’s analysis period overlaps with the COVID-19 pandemic, this may have affected the interpretation of study results and conclusions due to changes in healthcare-seeking behaviors and disrupted health services during this period [37, 38].

This study investigated OCS use pre-initiation of biologic therapy in patients with CRSwNP in Canada, providing clinically relevant perspectives on OCS use in this patient population. These results suggest that patients with CRSwNP are overexposed to OCS before the initiation of biologic therapies, which could lead to the increased risk of associated adverse effects. Enhancing physicians’ awareness of the appropriate use of OCS and the timely initiation of biologic therapies could reduce OCS-related adverse effects and improve disease management. Together, these results serve as a crucial step towards improving the decision-making process for physicians and, ultimately, patient outcomes.

## Supplementary Information

Additional file1 (DOCX 447 KB)

## Data Availability

The statements, findings, conclusions, views, and opinions expressed in this manuscript are based in part on data obtained under license from IQVIA Solutions Canada Inc. (IQVIA) concerning the following information service(s): IQVIA Private Drug Plan Database, May 2018–November 2023. All Rights Reserved. Permission to access such data must be sought out from IQVIA directly.

## Abbreviations

ACTIONS: ChAracterizing oral Corticosteroid use in patients wiTh chronic rhInOsinusitis with Nasal polypS in Canada
ALERT: ChAracterizing uncontroLled sevERe asThma in Canada
COPD: Chronic obstructive pulmonary disease
CRSwNP: Chronic rhinosinusitis with nasal polyps
GPFM: General practice and family medicine
INCS: Intranasal corticosteroids
OCS: Oral corticosteroids
PDP: Private drug plan
SD: Standard deviation
US: United States
