# Supplementary material for: Oral corticosteroid overexposure: characterizing oral corticosteroid use in patients with chronic rhinosinusitis with nasal polypS in Canada (ACTIONS) results
Source: Allergy Asthma Clin Immunol. 2025 Dec 4;21:51. doi: 10.1186/s13223-025-00989-w (PMC12687512; doi:10.1186/s13223-025-00989-w)
Supplement: Supplementary file 1 — Additional file1 (DOCX 447 KB) [file 13223_2025_989_MOESM1_ESM.docx]

**Supplement**

# Methods

## Study analyses

Selected patients with chronic rhinosinusitis with nasal polyps were further categorized into oral corticosteroid (OCS) burst or OCS maintenance sub-cohorts, or both, if they met certain requirements. Patients with ≥ 1 claim(s) with 3–28 days’ supply of ≥ 20 mg OCS (prednisone/equivalent) during the analysis period were classified into the OCS burst sub-cohort and those with ≥ 1 claim(s) with > 28 days’ supply of OCS during the analysis period into the OCS maintenance sub-cohort [1]. Patients were also categorized into asthma sub-cohorts based on previously published eligibility rules [2]. The overall asthma sub-cohort included all patients with respiratory products who were inferred as having an asthma diagnosis based on the rule-based inference algorithm. Patients from the asthma sub-cohort who did not meet the corresponding eligibility rules for severe asthma were categorized into the mild/moderate asthma sub-cohort; those who did meet the eligibility rules were included in the severe asthma sub-cohort. Patients who did not meet criteria of the aforementioned sub-cohorts were only included in the total study cohort.

# Results

Results were collected at a sub-provincial level. In Ontario, the median (interquartile range [IQR]) number of OCS claims per patient over the 24-month analysis period was 2.0 (4.0), with the highest number of claims reported in the central region at 3.0 (5.0). In Quebec, the median (IQR) number of OCS claims per patient was 4.0 (6.0), and the highest number of claims were reported in the grouped north region at 4.0 (6.0).

The median (IQR) number of physician specialties for OCS claims per patient in Ontario was 1.0 (1.0) and the highest average number of specialties for OCS claims occurred in the central region at 2.0 (1.0). The median (IQR) number of physician specialties for OCS claims per patient in Quebec was 1.0 (1.0) and both the grouped north region (1.0 [1.0]) and Montreal region (1.0 [1.0]) reported the highest average specialties for OCS claims.

Patients in Ontario with ≥ 1 OCS claim(s) had a median (IQR) total OCS dose per patient of 800 (1210) mg over the 24-month analysis period and 34% of patients had OCS overexposure in the 0–12 months, 13–24 months, or full 24-month period pre-index. In the Ontario province, the central region had the highest total median (IQR) OCS dose of 1180 (1850) mg in patients with ≥ 1 OCS claim(s) and 49% of patients had OCS overexposure. Patients in Quebec with ≥ 1 OCS claim(s) had a median (IQR) total OCS dose per patient of 1290 (1615) mg over the 24-month analysis period and 50% of patients had OCS overexposure in the 0–12 months, 13–24 months, or full 24-month period pre-index. In the Quebec province, the grouped north region had the highest total median (IQR) OCS dose of 1625 (1675) mg in patients with ≥ 1 OCS claim(s) and 60% of patients had OCS overexposure.

# References

1. Harvey J, Zhang S, Mu G, Valliant S, Alfonso-Cristancho R. CO34 characterization of OCS use in patients starting biologics for asthma in the United States. Value Health. 2023;26:S20.
2. Côté A, Beach J, Reynolds J, Usuba K, Tusin J, Tehrani A, et al. OCS use in uncontrolled severe asthma in Canada. Eur Respir J. 2024;64(Suppl. 68):PA4876.

**Supplementary Table 1** Demographics characteristics of OCS burst and maintenance sub-cohorts

|  | **OCS burst sub-cohort  (*n* = 581)** | **OCS maintenance sub-cohort (*n* = 135)** |
| --- | --- | --- |
| **Age, median (IQR)** | 52 (17) | 54 (18) |
| **Biological sex, (%)^a^** |  |  |
| Male | 294 (50.6) | 59 (43.7) |
| Female | 285 (49.1) | 74 (54.8) |
| **Private payer, (%)** | 581 (100) | 135 (100) |
| **Provinces in PDP database, (%)** |  |  |
| Alberta | 53 (9.1) | 6 (4.4) |
| British Columbia | 13 (2.2) | 3^b^ |
| Manitoba | 14 (2.4) | 3^b^ |
| New Brunswick | 19 (3.3) | 7 (5.2) |
| Newfoundland & Labrador | 3^b^ | 3^b^ |
| Nova Scotia | 15 (2.6) | 7 (5.2) |
| Ontario | 278 (47.8) | 59 (43.7) |
| Quebec | 178 (30.6) | 45 (33.3) |
| Saskatchewan | 3^b^ | 3^b^ |
| **Presence of comorbid AD, (%)** |  |  |
| Yes | 56 (9.6) | 12 (8.9) |
| No | 525 (90.4) | 123 (91.1) |
| **Medication use, (%)** |  |  |
| Antibiotic use | 496 (85.4) | 116 (85.9) |
| Inhaler use | 541 (93.1) | 127 (94.1) |
| INCS use | 409 (70.4) | 102 (75.6) |
| **Polypharmacy category****, (%)**^c^ |  |  |
| 1–4 | 49 (8.4) | 3^b^ |
| 5–7 | 160 (27.5) | 30 (22.2) |
| 8–10 | 154 (26.5) | 40 (29.6) |
| 11–13 | 117 (20.1) | 23 (17.0) |
| 14–16 | 55 (9.5) | 23 (17.0) |
| 17–19 | 26 (4.5) | 3^b^ |
| 20+ | 20 (3.4) | 12 (8.9) |

^a^Sum of values may equal 100% as some data were unknown.
^b^All values with a count of less than six patients or claims were masked as three according to privacy rules including secondary values.
^c^Polypharmacy was defined as the number of EphATC (level 2) classes in any market in the 24-month analysis period; patients with unknown polypharmacy had the ATC information unavailable in the 24-month analysis period.
AD, atopic dermatitis; (Eph)ATC, (European Pharmaceutical) Anatomical Therapeutic Chemical classification; INCS, intranasal corticosteroid; IQR, interquartile range; OCS, oral corticosteroids; PDP, Private Drug Plan.

**Supplementary Table 2** OCS use at sub-provincial level

|  | **Province** | | | | | | | | |
| --- | --- | --- | --- | --- | --- | --- | --- | --- | --- |
|  | **AB** | **BC** | **MB** | **NB** | **NL** | **NS** | **ON** | **QC** | **SK** |
| **Patients, *n*** | 66 | 26 | 25 | 20 | 3^a^ | 21 | 364 | 213 | 3^a^ |
| **Number of OCS claims, *n* (%)** |  |  |  |  |  |  |  |  |  |
| 0 | 11 (16.7) | 12 (46.2) | 8 (32.0) | 3^a^ | — | 3^a^ | 76 (20.9) | 29 (13.6) | — |
| 1–2 | 29 (43.9) | 3^a^ | 9 (36.0) | 3^a^ | — | 3^a^ | 127 (34.9) | 54 (25.4) | — |
| > 2 | 26 (39.4) | 3^a^ | 8 (32.0) | 13 (65.0) | — | 15 (71.4) | 161 (44.2) | 130 (61.0) | — |
| **Patients with ≥ 1 OCS claim(s),  *n* (%)** | 55 (100) | 14 (100) | 17 (100) | 19 (100) | — | 16 (100) | 288 (100) | 184 (100) | — |
| **Median total daily dose of OCS  per patient, mg (IQR) in those with ≥ 1 OCS claim(s)** | 620 (1240) | 1120 (713) | 1100 (1475) | 1335 (1585) | — | 1775 (2747) | 800 (1210) | 1290 (1615) | — |
| **Median total number of days of OCS receipt per patient, *n* (IQR) in those with ≥ 1 OCS claim(s)** | 23 (35) | 54 (37) | 28 (89) | 42 (74) | — | 63 (163) | 28 (49) | 42 (69) | — |

^a^All values with a count of less than six patients or claims were masked as three according to privacy rules including secondary values.
AB, Alberta; BC, British Columbia; IQR, interquartile range; MB, Manitoba; NB, New Brunswick; NL. Newfoundland and Labrador; NS, Nova Scotia; OCS, oral corticosteroids; ON, Ontario; QC, Quebec; SK, Saskatchewan.

**Supplementary Fig. 1** Study design
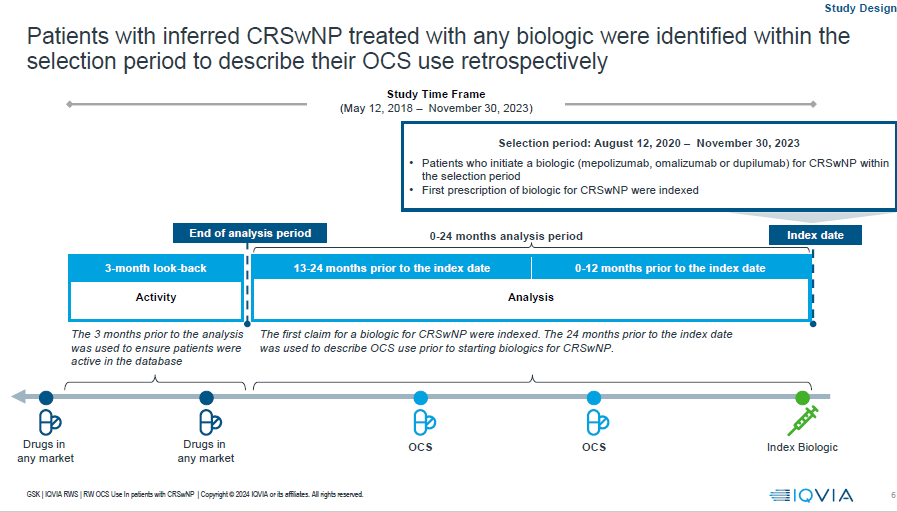


CRSwNP, chronic rhinosinusitis with nasal polyps; OCS, oral corticosteroids**.**

**Supplementary Fig. 2** Rule-based indication algorithms for mepolizumab, omalizumab, or dupilumab patients

**
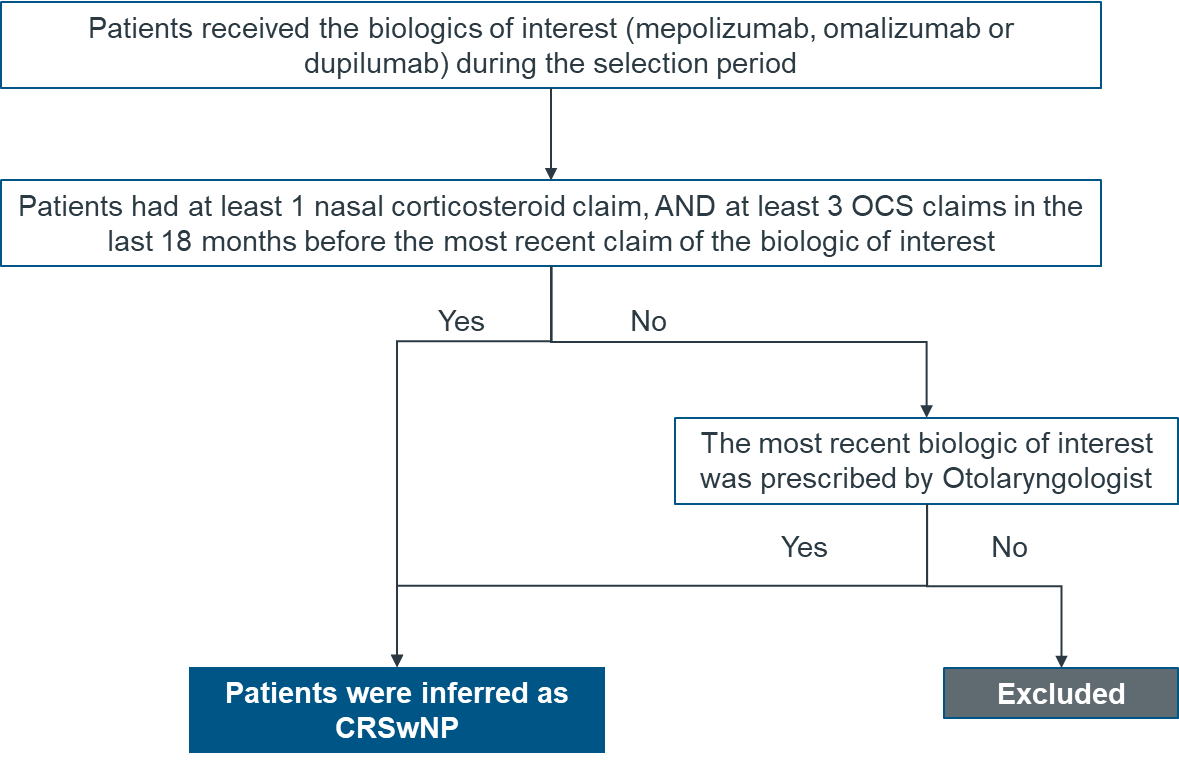
**

CRSwNP, chronic rhinosinusitis with nasal polyps; OCS, oral corticosteroid.

**Supplementary Fig. 3** Proportion of patients with OCS claims in the OCS burst and maintenance sub-cohorts


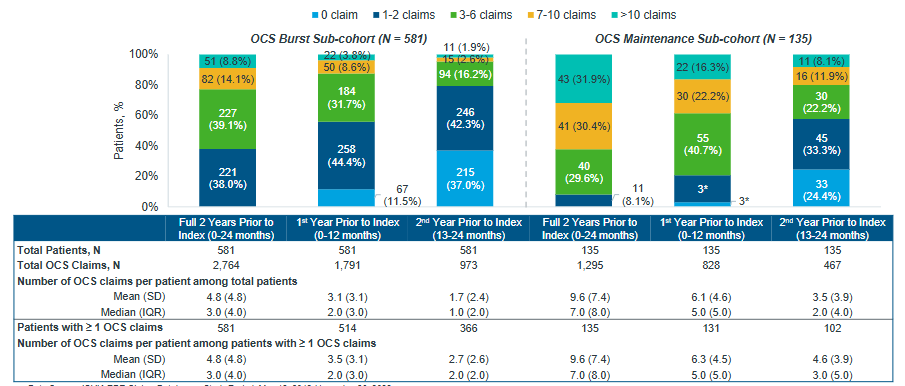


*All values with a count of less than six patients or claims were masked as three according to privacy rules including secondary values.
IQR, interquartile range; OCS, oral corticosteroids; SD, standard deviation.

**Supplementary Fig. 4** OCS doses received by patients in the OCS burst sub-cohort in the full 24-month analysis period (**A**), and 0–12 months and 13–24 months pre-biologic therapy initiation (**B**)

**A**


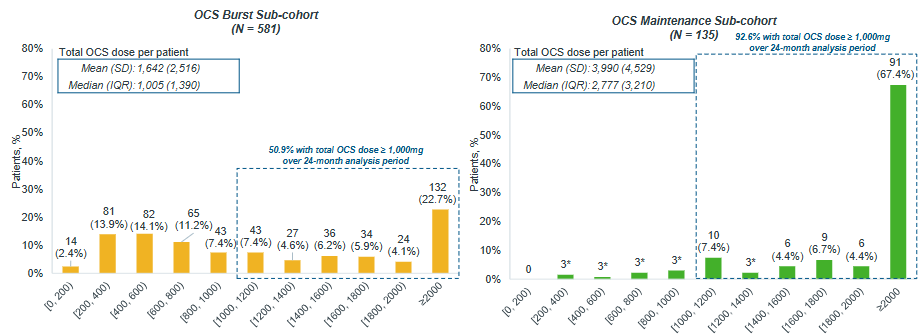


**B**


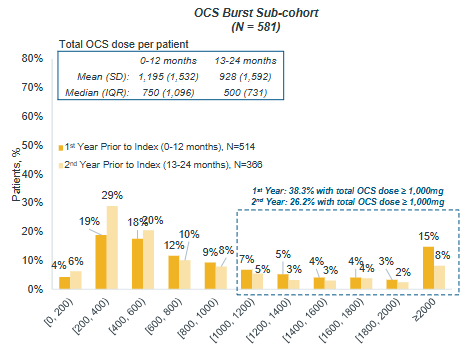


IQR, interquartile range; OCS, oral corticosteroids; SD, standard deviation.

**Supplementary Fig. 5** OCS doses received by patients in the OCS maintenance sub-cohort in the full 24-month analysis period (**A**), and 0–12 months and 13–24 months pre-biologic therapy initiation (**B**)

**A**


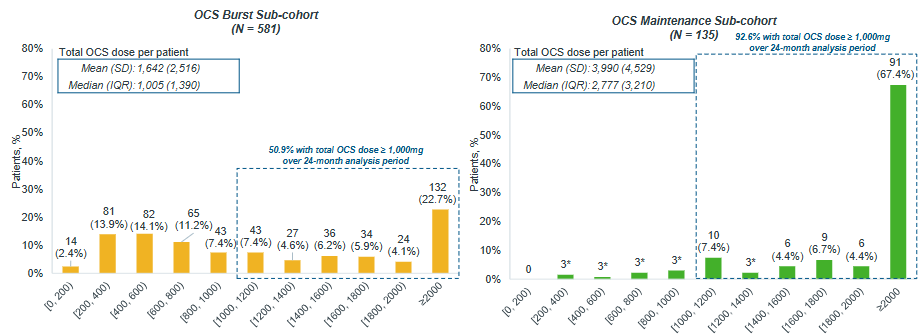


**B**


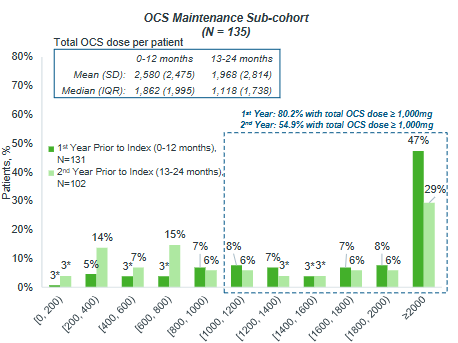


*All values with a count of less than six patients or claims were masked as three according to privacy rules including secondary values.
IQR, interquartile range; OCS, oral corticosteroids; SD, standard deviation.

**Supplementary Fig. 6** Proportion of OCS claims by physician specialty in the OCS burst and maintenance sub-cohorts


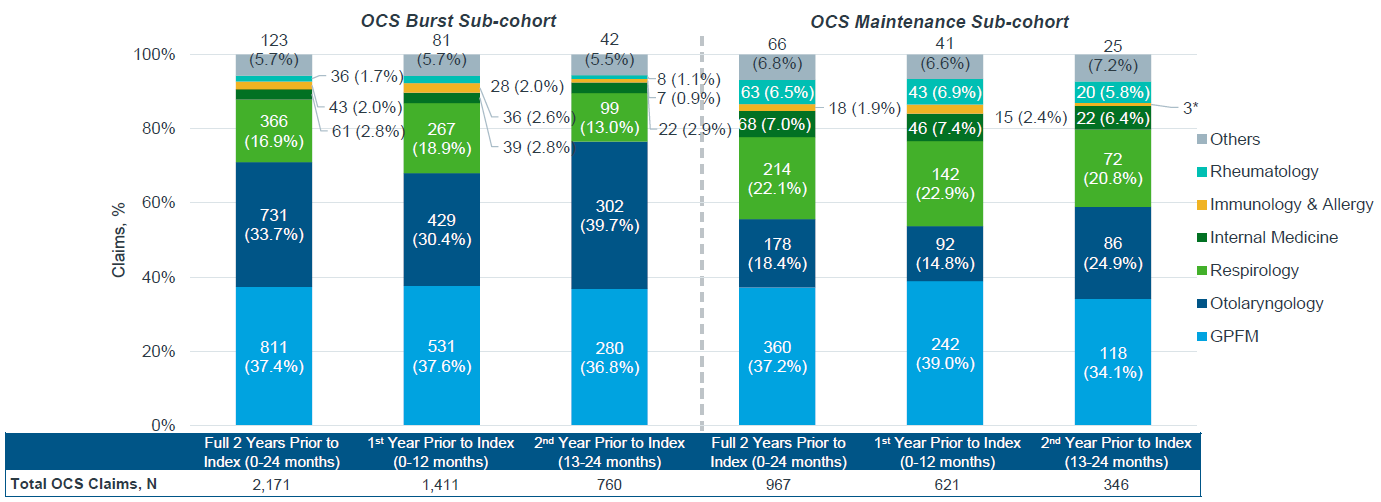


*All values with a count of less than six patients or claims were masked as three according to privacy rules including secondary values.
GPFM, general practice and family medicine; OCS, oral corticosteroids.
